# A Call to Action: Building a Translational Inclusion Team Science in Physical Activity, Nutrition, and Obesity Management for Children with Disabilities

**DOI:** 10.3389/fpubh.2016.00164

**Published:** 2016-08-10

**Authors:** James H. Rimmer, Kerri A. Vanderbom

**Affiliations:** ^1^University of Alabama at Birmingham/Lakeshore Foundation Research Collaborative, Birmingham, AL, USA; ^2^Department of Physical Therapy, University of Alabama at Birmingham, Birmingham, AL, USA

**Keywords:** health promotion and disease prevention, community health inclusion, people with disability, evidence-based practice, guideline and program adaptation

## Abstract

The growing evidence base of childhood obesity prevention and treatment programs do not adequately consider how to adapt these programs for children with disabilities. We propose a *Call to Action* for health researchers who conduct studies focused on the general population (i.e., without a disability) to work closely with disability researchers to adapt their programs (e.g., obesity management, increased physical activity, and caregiver training in diet and nutrition) to be relevant to both groups. We refer to this approach as inclusion team science. The hope for this *Call to Action* is that there will be greater synergy between researchers who have high levels of expertise in a specialty area of health (but little or no knowledge of how to adapt their program for children with disabilities) to work more closely with researchers who have a high level of expertise in adapting evidence-based health promotion recommendations and strategies for children with disabilities. Together, these two areas of expertise will lead to inclusive physical activity and nutrition programs for all children.

Health-promoting activities have a particularly important value for children with disabilities because of their higher rates of sedentary behavior and greater risk of disability-associated secondary health conditions (1, 2). Increased physical activity can have an enormous impact on reducing secondary conditions and improving the health of children with disabilities (3). Benefits include improvements in gross motor function (4, 5), prevention of deconditioning (6), and increased physical independence (7). These effects are augmented with the presence of good nutrition.

In the current environment, children with disabilities face enormous challenges in acquiring health behaviors (i.e., physical activity and nutrition) critical to weight management and optimization of health. They are much less likely to participate in school and community-based health promotion programs, far more likely to be sedentary, and have a poorer nutritional status (8–11). Functional limitations associated with a physical or cognitive disability can result in a difficult interaction between the child and environment. Inaccessible facilities, lack of transportation to and from indoor and outdoor recreation venues, absence of knowledgeable staff who know how to adapt programs, and a general perception/attitude among providers that children with disabilities need “specialized” vs. integrated services feeds into a culture of isolation and separation (12–14). When these barriers are juxtaposed with the lack of interest, awareness or understanding among service providers regarding how and why they should include children with disabilities in mainstreamed health promotion programs, a vicious cycle is activated that begins with restricted access to physical activity and nutrition education; this leads to a greater number of health problems associated with sedentary behavior and poor diet; and finally, more health problems result in further isolation from peers without disability and a greater vulnerability to early onset health disparities (12, 15–17).

As illustrated in Figure 1, health promotion programs for the general population of children and specialized programs for children with disabilities currently tend to be developed and delivered within separate spheres of activity. The left side of the figure shows how this parallel structure may result in inefficient use of resources and inadvertently promote practices and programs that never intersect. While specialized health promotion programs for children with disabilities are quite valuable in situations where a child desires or needs to participate in sports- or disability-specific opportunities (e.g., wheelchair basketball and Special Olympics) to learn and practice specific skills, for example, these programs often have limited availability and frequency (i.e., many are only offered 1 or 2 days per week). There is a pressing need to provide greater amounts of access to mainstreamed physical activity and nutrition programs offered in schools, healthcare facilities, community-based organizations, and outdoor recreation areas. The right side of the figure illustrates the potential benefit of a more *inclusive* framework that supports both children with and without disabilities, but does not negate the need for specialized programs offered to children interested or needing certain services that cannot be provided in mainstreamed settings.

**Figure 1:**
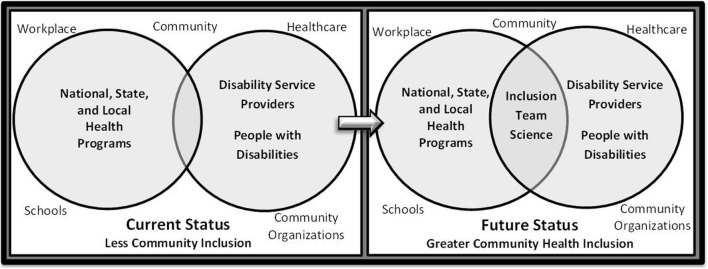
**Current and Future Status of Community Health Inclusion**.

## A Call to Action: Building an Inclusion Team Science That Focuses on Environmental and Program Adaptation vs. Reinvention

To more effectively prevent and control childhood obesity and optimize health, the Institute of Medicine (18), Centers for Disease Control and Prevention (19), and National Institutes of Health have begun to promote multilevel (e.g., family, organization, and community), multisector (e.g., family, school, health care, community, and policy) approaches that focus on changing, not just individual behavior, but also addressing the broader sociophysical environment in which children live, learn, eat, and play (20). Children with disabilities must be given the opportunity to be included in these initiatives in the same environmental ecosystems that children without disabilities use to engage in positive health behaviors (e.g., outdoor and indoor play, recreation and sport; improved nutritional guidance in homes, schools, and clinics) in order to gain the same health benefits.

One way to disentangle the separate research agendas in health promotion between children with and without disability is to encourage adaptation of successful, evidence-based programs established for children without disability (often referred to as translation research). The Guidelines International Network defines guideline adaptation as “*the systematic approach to the endorsement and/or modification of a guideline(s) produced in one cultural and organizational setting for application in a different context. Adaptation may be used as an alternative to de novo guideline development, e.g., for customizing (an) existing guideline(s) to suit the local context*.” (21) There are several benefits to guideline or program adaptation (22–24): (1) reduces duplication of effort while maintaining the validity of evidence-based recommendations, (2) encourages a participative approach involving key stakeholders in order to foster local ownership of recommendations and promote utilization, (3) ensures consideration of (regional and local) contextual factors to improve uptake by targeted users, and (4) improves guideline/program quality by increasing knowledge and commitment to evidence-based principles using reliable methods to ensure quality and validity of adapted guidelines/programs and promotes explicitness and transparency in documenting recommendations.

A recent methodology has been developed that allows researchers and programmers to adapt evidence-based guidelines and programs in physical activity and nutrition for adults and children with disabilities (25). The tool/method is referred to as the GRAIDs Framework, which stands for *G*uidelines, *R*ecommendations, *A*daptations *I*ncluding *D*isability. The GRAIDs Framework is a systematic process for obtaining collaborative information from a coalition of experts in the field as well as individuals with disabilities and their families. The GRAIDs Framework has, thus, far been used to adapt CDC’s evidence-based obesity prevention strategies (19) to be inclusive of children and adults with disabilities. Utilization of the GRAIDS framework has the potential to provide children with disabilities and their caregivers with timely and suitable guideline/program adaptations for physical activity and nutrition that will afford them the ability to actively participate in inclusive programs with their non-disabled peers.

## Adaptation Example: The Brief Motivational Interviewing Project (BMI^2^)

The BMI^2^ (Brief Motivational Interviewing to reduce BMI) study tested the impact of motivational interviewing (MI) delivered by primary care providers and registered dietitians (RD) on pediatric obesity in a non-disabled population (26). The target group was parents of overweight children of ages 2–8 years. Forty-two practices from the American Academy of Pediatrics, Pediatric Research in Office Settings Network were randomly assigned to one of the three groups. Group 1 (usual care) measured BMI percentile at baseline, 1-year, and 2-year follow-up. Group 2 (provider only) delivered 4 MI counseling sessions to parents of the participating child over 2 years. Group 3 (Provider + RD) delivered 4 provider MI sessions plus 6 MI sessions from a RD. The primary outcome was child BMI percentile at 2-year follow-up. At 2-year follow-up, the adjusted BMI percentile was 90.3, 88.1, and 87.1 for Group 1, 2, and 3, respectively. The Group 3 mean was significantly (*p* = 0.02) lower than Group 1. Mean changes from baseline in BMI percentile were 1.8, 3.8, and 4.9 across Groups 1, 2, and 3. MI delivered by providers and RDs (Group 3) resulted in statistically significant reductions in BMI percentile.

### Adapting the BMI^2^ Program for Children with Disabilities

The GRAID Framework was used to develop an inclusive version of BMI^2^ and is comprised of several inclusion recommendations and adaptations that relate to providing education and training to program staff who are not knowledgeable in working with families who have a child with a disability and who may have varying levels of physical and cognitive function. A brief example of a GRAID developed for the BMI^2^ program can be found in Table 1. Each guideline/program applied to the GRAID Framework has a menu of inclusion recommendations and adaptations that allow the provider to select the ones that are relevant to their local context and need. Adaptations can be tested in an iterative nature and, when found effective, can be cataloged for future use with other families who have similar needs. A unique feature of the adapted guidelines/programs are the *inclusion elements*, print and video resources that are linked to each adaptation and offered through the National Center on Health, Physical Activity, and Disability (www.nchpad.org). The inclusion elements are examples of successful applications of the adaptations in real life settings.

**Table 1:** **An example of GRAID inclusion recommendations and adaptations for an evidence-based weight management program (BMI^2^) (26)**.

Guideline: healthcare providers should include children with disabilities in health promotion programs	
(1) Inclusion recommendation	Healthcare facilities should educate healthcare professionals about disability, obesity, and health
(a) Adaptation	Offer a training session about disability and the problems related to obesity, how to prevent and treat obesity, and where to find data on the topic
(2) Inclusion recommendation	Healthcare facilities should train healthcare professionals about strategies to increase physical activity for children with disabilities
(a) Adaptation	Educate doctors and RDs about setting appropriate physical activity goals for children with disabilities (e.g., importance of self-discovery, decision making and choice, and independence)
(3) Inclusion recommendation	Healthcare facilities should train healthcare professionals about policies supporting the participation of children with disabilities in all aspects of their community
(4) Inclusion recommendation	Health promotion programs should develop and disseminate educational materials inclusive of children with disabilities representing diverse ethnic and racial backgrounds
(a) Adaptation	Incorporate inclusive images of children with disabilities and terminology representing diverse ethnic and racial backgrounds in physical activity and healthy nutrition educational materials
(b) Adaptation	Provide physical activity and nutrition educational materials in accessible, linguistically appropriate formats (e.g., larger font, web-accessible, in the preferred language)

## Building an Inclusion Team Science to Avoid “Reinventing the Wheel”

Concern over the rapidly increasing incidence and prevalence of health disparities among people with disabilities (27) has produced intense interest among federal agencies in identifying evidence-based strategies and practices to prevent or reduce these disparities. Ideally, the “evidence base” from which such guidelines or strategies would be derived would consist of rigorously conducted empirical studies with appropriate representation of all target populations in the data. Unfortunately, there are few areas of disability health or rehabilitation research in which the sample size and scientific rigor of studies compares favorably with that typically found in large scale general population studies.

Federal agencies have recently recognized this deficit and increasingly have funding opportunities that call for coordinated teams of investigators with diverse skills and knowledge to conduct studies of complex social problems with multiple causes and etiologies who can work toward a common health goal. In the case of disability, the ideal environment would be to target an area of health (e.g., physical inactivity or obesity) that would involve two studies: the primary study would be directed at the larger target population, which in the case of the BMI^2^ study involved children and their families, and the adjunct study would address a subgroup of children with disabilities. What this would allow for is the interaction of experts in obesity research blending with experts in disability who can connect these two areas of science.

Toward that end, the hallmark of team science is collaboration to address a scientific challenge that leverages the strengths and expertise of professionals trained in different fields. This allows for such problems to be examined from multiple perspectives, ultimately giving rise to comprehensive and integrative solutions and minimizing duplication of effort and reinventing the wheel.

Inasmuch as researchers are accustomed to working within their respective areas of expertise, consideration must be given to the organization, composition, and dynamics of the team. Scientific leadership must ensure that all perspectives are equitably included in the design and conduct of the study and that the structure and organization of the team facilitates meaningful involvement of all parties. This is especially important for teams that engage multiple stakeholders, including community members, service providers, and policymakers. Each perspective contributes to the team’s ability to achieve a common health goal and to foster the translation of study findings to practice and policy.

## Conclusion

Public health programs and professionals who work in schools, fitness and recreation centers, and healthcare facilities must recognize the low rates of physical activity participation and poor nutrition among people with disabilities and begin to develop effective and cohesive strategies to address this problem. While most of the financial resources in public health have been directed at prevention of disease, injury, and disability, there is growing recognition among public policy experts that prevention of secondary conditions is an equally important issue among people with disabilities. Health promotion activities, especially increased participation in physical activity and improved nutrition, can have an enormous positive impact on reducing secondary conditions and improving health in children with disabilities.

While there will always be a need for specialized research and programs targeting specific subgroups (i.e., children with physical/cognitive disability), a model that begins with inclusion team science can serve as the foundation for building a framework that uses the successful elements of adaptation (i.e., GRAIDs) for promoting inclusion in existing and new programs.

Implementing new evidence-based research findings that are in the early stages of development could take years, or perhaps decades, to reach application in clinical or community practice (28). Use of the GRAIDs Framework in future research and programmatic efforts provides a unique opportunity to test their utility in mainstreamed health promotion programs and build a database of practice-based evidence. Successful adaptations can then be cataloged and scaled to other communities, with the intention to keep children with disabilities and their family members an integral part of an inclusive, supportive community.

## Author Contributions

JR conceived the topic and focus of this article. Both JR and KV contributed to the writing and content.

## Conflict of Interest Statement

The authors declare that the research was conducted in the absence of any commercial or financial relationships that could be construed as a potential conflict of interest.
